# Gastroesophageal Junction and Pylorus Distensibility Before and After Sleeve Gastrectomy—pilot Study with EndoFlip^TM^

**DOI:** 10.1007/s11695-023-06606-2

**Published:** 2023-04-28

**Authors:** Christian Tibor Josef Magyar, Yves Borbély, Reiner Wiest, Guido Stirnimann, Daniel Candinas, Johannes Lenglinger, Philipp C. Nett, Dino Kröll

**Affiliations:** Department of Visceral Surgery and Medicine, Inselspital, Bern University Hospital, University of Bern, 3010 Bern, Switzerland

**Keywords:** Sleeve gastrectomy (SG), Gastroesophageal reflux disease (GERD), Impedance planimetry, Endoluminal functional lumen imaging probe (EndoFlip), Distensibility index (DI), Pylorus, Gastroesophageal junction (GEJ)

## Abstract

Sleeve gastrectomy (SG) is the most frequently performed bariatric surgical intervention worldwide. Gastroesophageal reflux disease (GERD) is frequently observed after SG and is a relevant clinical problem. This prospective study investigated the gastroesophageal junction (GEJ) and pyloric sphincter by impedance planimetry (EndoFlip^TM^) and their association with GERD at a tertiary university hospital center. Between January and December 2018, patients undergoing routine laparoscopic SG had pre-, intra-, and postoperative assessments of the GEJ and pyloric sphincter by EndoFlip^TM^. The distensibility index (DI) was measured at different volumes and correlated with GERD (in accordance with the Lyon consensus guidelines). Nine patients were included (median age 48 years, preoperative BMI 45.1 kg/m^2^, 55.6% female). GERD (de novo or stable) was observed in 44.4% of patients one year postoperatively. At a 40-ml filling volume, DI increased significantly pre- vs. post-SG of the GEJ (1.4 mm^2^/mmHg [IQR 1.1–2.6] vs. 2.9 mm^2^/mmHg [2.6–5.3], *p* VALUE=0.046) and of the pylorus (6.0 mm^2^/mmHg [4.1–10.7] vs. 13.1 mm^2^/mmHg [7.6–19.2], *p* VALUE=0.046). Patients with postoperative de novo or stable GERD had a significantly increased preoperative DI at 40 ml of the GEJ (2.6 mm^2^/mmHg [1.9–3.5] vs. 0.5 mm^2^/mmHg [0.5–1.1], *p* VALUE=0.031). There was no significant difference in DI at 40 mL filling in the preoperative pylorus and postoperative GEJ or pylorus. In this prospective study, the DI of the GEJ and the pylorus significantly increased after SG. Postoperative GERD was associated with a significantly higher preoperative DI of the GEJ but not of the pylorus.

**Graphical Abstract:**

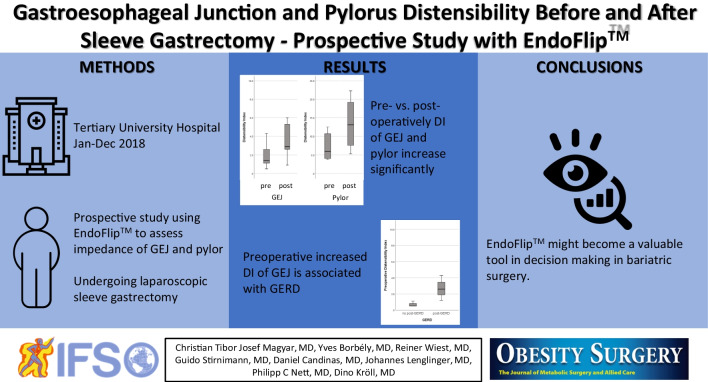

**Supplementary Information:**

The online version contains supplementary material available at 10.1007/s11695-023-06606-2.

## Introduction

A major drawback of sleeve gastrectomy (SG) is the postoperative development of “de novo” or worsening gastroesophageal reflux disease (GERD) [1–4]. GERD is considered a risk factor for long-term complications and decreases the quality of life [5–7]. The gastroesophageal junction (GEJ) (often also referred to as the lower esophageal sphincter) plays an essential role in the complex pathophysiology of GERD, and a better understanding of the associated anatomical and functions is necessary. To date, the usefulness of an endoscopic functional luminal imaging probe (EndoFlip™) in bariatric patients before and after SG to assess the pathophysiological changes in the gastroesophageal junction (GEJ) and pylorus with regard to GERD is poorly described. We hypothesized that SG leads to increased distensibility indices (DI) of the GEJ and the pylorus.

## Materials and Methods

This prospective diagnostic observational study included patients (informed consent, ≥18 years, BMI ≥35 kg/m^2^, fulfillment of the Swiss Society for the Study of morbid obesity and metabolic disorders guidelines criteria) undergoing elective SG at a tertiary care university hospital. The study was approved by the local ethics committee (BASEC ID 2017-00923). Exclusion criteria were pregnancy or breastfeeding, oral anticoagulant, known bleeding disorder, and contraindication for endoscopic examination. Patients underwent esophago-gastro-duodenoscopy (EGD), pH-impedance monitoring, high-resolution manometry, and EndoFlip™ (Crospon Medical Devices, Galway, Ireland). GERD was defined (Lyon Consensus 2018) as distal oesophageal acid (pH<4) exposure time >6% and a total number of reflux episodes >80 (as per pH-impedance monitoring) [8] and was differentiated between no, stable, de novo, or resolved status when comparing pre and postoperative data. Bariatric outcomes were reported in accordance with the American Society for Metabolic and Bariatric Surgery (ASMBS) guidelines [9]. Statistical analysis (categorical by Pearson chi-square test and continuous by Mann–Whitney ***U*** test) was performed using SPSS Statistics version 25 (IBM Corporation, Armonk, New York).

## Results

Between January 1, 2018, and December 31, 2018, nine patients (Table 1) with complete pre- and postoperative EndoFlip™ data were prospectively enrolled in this pilot study. No relevant hiatal hernias were documented.

**Table 1:** Demographics, clinical characteristics, and bariatric and surgical outcomes

	Total
	*n*=9
Age [years]	48 (46–56)
Gender [female]	5 (55.6%)
Body mass index [kg/m^2^]	45.1 (39.7–49.9)
Comorbidities	
Diabetes mellitus	4 (44.4%)
Chronic heart failure or coronary artery disease	1 (11.1%)
Arterial hypertension	6 (66.7%)
OSAS	7 (77.8%)
Chronic kidney disease	2 (22.2%)
NASH/NAFLD	4 (44.4%)
Psychiatric disease	2 (22.2%)
Nicotine abuse	2 (22.2%)
Bariatric and surgical outcome	
Length of hospital stay [days]	2.9 (2–3)
1-year follow-up BMI [kg/m^2^]	32.3 (29.4–36)
%TWL [%]	28.7 (24.2–29.2)
%EWL [%]	102.2 (69.9–106.7)
%EBMIL [%]	69.5 (55.9–68.1)
Delta BMI [kg/m^2^]	12.8 (10.3–13.7)
Median length of follow-up [months]	25.2 (14–37)
Postoperative GERD	
GERD “de novo”	2 (33.3%)
GERD resolution	3 (33.3%)
GERD stable	2 (22.2%)
No GERD	1 (11.1%)
Postoperative PPI use at 1 year	4 (44.4%)

Values are medians (interquartile ranges (IQR)) or number (percentages), respectively

Abbreviations: *%EBMIL*, percentage of excess of BMI loss; *%EWL*, percentage of excess weight loss; *%TWL*, percentage of total weight loss; *BMI*, body mass index; *GERD*, gastroesophageal reflux disease; *ICU*, intensive care unit; *NAFLD*, nonalcoholic fatty liver diseases; *NASH*; nonalcoholic steatotic hepatitis; *OSAS*, obstructive sleep apnea syndrome; *PPI*, proton pump inhibitor

**Fig. 1:**
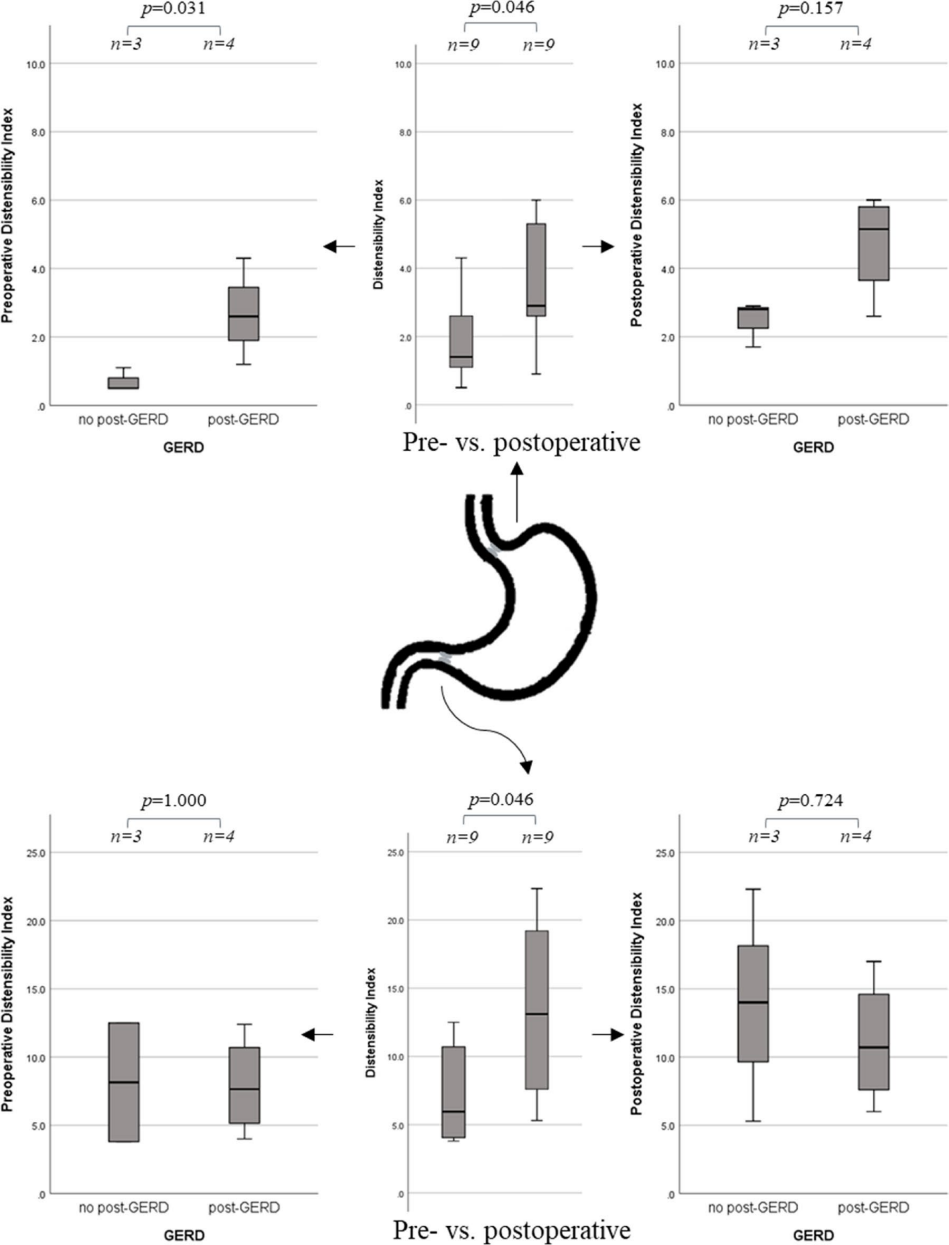
Analysis of the distensibility index (in mm^2^/mmHg) at 40mL filling volume, using EndoFlip^TM^, of the gastroesophageal junction and the pyloric sphincter comparing the pre- vs postoperative setting as well as for an association with 1-year postoperative gastroesophageal reflux disease. Abbreviations: GERD gastroesophageal reflux disease

EndoFlip^TM^ at 40 ml the DI of the GEJ was significantly higher post-SG compared to the presurgical assessment (1.4 mm^2^/mmHg [1.1-2.6] vs. 2.9 mm^2^/mmHg [2.6–5.3], *p* value=0.046) (Table 2, Fig. 1, and Suppl. Table 2a & b). Pylorus DI significantly increased post-SG (6.0 mm^2^/mmHg [4.1–10.7] vs. 13.1 mm^2^/mmHg [7.6–19.2], *p* value=0.046).

**Table 2:** Summary of the assessment of the lower esophageal sphincter and pylor in the pre-, intra-, and postoperative setting in sleeve gastrectomy

	Filling [ml]	Parameter	Preoperative	Intraoperative	Postoperative	∆ 95%CI pre-post	*p* value
GEJ	40	D_Min_ [mm]	9.4 (6.9–12)	13.7 (11.5–18.2)	11.3 (7.8–15.2)	1.6 (−1.9–5.1)	0.401
		DI [mm^2^/mmHg]	1.4 (1.1–2.6)	3.8 (1.9–8.9)	2.9 (2.6–5.3)	1.6 (−0.5–3.2)	0.046
Pylorus	40	D_Min_ [mm]	13.4 (12.6–16.9)	13.7 (12.5–17.2)	16.2 (14.6–17.3)	1.2 (−1.9–4.4)	0.401
		DI [mm^2^/mmHg]	6.0 (4.1–10.7)	4.1 (2.3–9.9)	13.1 (7.6–19.2)	6.2 (0.4–12.0)	0.046

Values are medians (interquartile ranges (IQR)), differences given as mean with 95%CI; significances assessed using Mann-Whitney *U comparing pre- and postoperative values*.

Abbreviations: ∆, delta or difference; *DI*, distensiblity index; *D*_*Min*_, minimal diameter; *GERD*, gastroesophageal reflux disease

### Gastroesophageal Reflux Disease

The post-GERD (pGERD) group (de novo or stable GERD, *n*=4/9) was compared to the no post-GERD (npGERD) group (resolution of or no GERD, *n*=3/9). Two patients with insufficient GERD data (one conversion to gastric bypass and one without pH-impedance monitoring) were excluded from further analysis. Neither %EWL (*p* value=0.229), %TWL (*p* value=0.857), %EBMIL (*p* value=0.229), nor delta BMI (*p* value=0.114) at 1 year were significantly different between pGERD and npGERD.

Preoperatively obtained DI (40 ml) of the GEJ were significantly different between the pGERD group and npGERD group (2.6 mm^2^/mmHg [1.9–3.5] vs. 0.5 mm^2^/mmHg [0.5–1.1], *p* value=0.031) (Tables 3, 4 and Fig. 1). No significant difference in the pyloric DI (40 ml) was found (*p* value=1.000). Intraoperatively, the DI (40 ml) was not significantly different between pGERD and npGERD for the GEJ (*p* value=0.248) or for the pylorus (*p* value=0.564). Postoperatively, the DI of GEJ nor pylorus was not significantly different between groups (*p* value=0.157, *p* value=0.724) (Table 4).

**Table 3:** Summary of the assessment for GERD of the lower esophageal sphincter and pylor in a preoperative setting in sleeve gastrectomy

	Filling [ml]	Parameter	Preoperative EndoFlip^TM^			
			Post-GERD	No post-GERD	∆ (95%CI)	*p* value
GEJ	40	D_Min_ [mm]	11.9 (10.1–12.5)	6.9 (5.9–6.9)	4.8 (1.8–7.7)	0.032
		DI [mm^2^/mmHg]	2.6 (1.9–3.5)	0.5 (0.5–1.1)	2.0 (0.4–3.9)	0.031
Pylorus	40	D_Min_ [mm]	15.1 (12.7–19.0)	14.1 (11.6–16.6)	1.8 (−7.5–11.1)	0.355
		DI [mm^2^/mmHg]	7.7 (5.2–10.7)	8.2 (3.8–12.5)	−0.2 (−10.8–10.3)	1.000

Values are medians (interquartile ranges (IQR)), differences given as mean with 95%CI; significances assessed using Mann-Whitney *U*

Abbreviations: ∆, delta or difference; *DI*, distensiblity index; *D*_*Min*_, minimal diameter; *GERD*, gastroesophageal reflux disease

**Table 4:** Summary of the assessment for GERD of the gastroesophageal junction and pylor in a postoperative setting in sleeve gastrectomy

	Filling [ml]	Parameter	Postoperative EndoFlip^TM^			
			Post-GERD	No post-GERD	∆ (95%CI)	*p*-value
GEJ	40	D_Min_ [mm]	13.9 (10.2–15.7)	9.0 (4.9–11.3)	4.5 (−2.5–11.5)	0.157
		DI [mm^2^/mmHg]	5.2 (3.7–5.8)	2.8 (1.7–2.9)	2.2 (−0.19–4.7)	0.157
Pylorus	40	D_Min_ [mm]	15.7 (13.0–17.3)	15.8 (14.7–16.5)	−0.5 (−4.9–3.8)	1.000
		DI [mm^2^/mmHg]	10.7 (7.6–14.6)	14.0 (5.3–22.3)	−2.7 (−20.4–14.8)	0.724

Values are medians (interquartile ranges (IQR)), differences given as mean with 95%CI; significances assessed using Mann-Whitney *U*

Abbreviations: ∆, delta or difference; *DI*, distensiblity index; *D*_*Min*_, minimal diameter; *GERD*, gastroesophageal reflux disease

## Discussion and Conclusion

This is the first prospective study assessing pre-, intra-, and 1-year postoperative changes in the GEJ and the pylorus using EndoFlip™ in SG. To evaluate GERD in SG, EndoFlip™ is a promising device for GEJ assessment to predict postoperative long-term functional outcomes. Normative values are required to improve the interpretation of EndoFlip™ in clinical practice. This study showed a significant difference in the DI (at 40 ml filling) of the GEJ by EndoFlip™ pre- vs. 1 year after SG. Preoperative increased DI of the GEJ is associated with GERD 1 year post-SG.

Our perioperative findings are in line with Reynolds et al. and Greenberg et al. [10–12] who argued that destructed sling fibers at the angle of His are to be regulated. However, these studies were performed in the operating room before and after stapler administration for the SG. We present the first 1-year postoperative data.

The development of GERD after SG may be a dynamic process [13]. Several mechanisms have been proposed which may lead to the development and resolution of GERD after SG (reduced gastric compliance, increased gastric pressure, shape of SG incl. preservation of antrum, delayed gastric emptying, pylorospasm, %TWL, hiatal hernia, and GEJ complex) [13]. However, no significant correlation was found between weight loss and change of GEJ measurements in the respective GERD groups.

Contrary to findings from Desprez et al., our results showed a significant increase in the DI of the pyloric sphincter from pre- to 1 year after SG, which could be associated with accelerated gastric emptying [14].

A main limitation is that our study consisted of a patient cohort and was insufficiently powered to allow in-depth analysis for the prediction of GERD after SG. The findings of this pilot study suggesting the usefulness of Endoflip™ to predict GERD after SG needs to be verified in larger studies.

## Supplementary information

ESM 1 (DOCX 20.6 KB)

## Data Availability

Upon request.
