# Supplementary material for: Gastroesophageal Junction and Pylorus Distensibility Before and After Sleeve Gastrectomy—pilot Study with EndoFlipTM
Source: Obes Surg. 2023 Apr 28;33(7):2255–60. doi: 10.1007/s11695-023-06606-2 (PMC10289900; doi:10.1007/s11695-023-06606-2)
Supplement: Supplementary file 1 — (DOCX 20.6 KB) [file 11695_2023_6606_MOESM1_ESM.docx]

|  |  | Total |
| --- | --- | --- |
|  |  | n=9 |
| Preoperative distal esophageal acid (ph<4) exposure time [%] | | 16.7 (4 - 9.5) |
| Preoperative total number of reflux episodes (acid and non-acid) | | 45 (34.5 - 58.5) |
| Preoperative manometry DCI mean | | 4479.8 (3626 - 5841) |
| Preoperative EGD Los Angeles Classification | None | 3 (33.3%) |
|  | A | 4 (44.4%) |
|  | B | 2 (22.2%) |
| Postoperative distal esophageal acid (ph<4) exposure time [%] | | 15.4 (1.2 - 14.3) |
| Postoperative total number of reflux episodes (acid and non-acid) | | 69.6 (35 - 109.5) |
| Postoperative manometry DCI mean | | 3267.6 (1545 - 4439) |
| Postoperative EGD Los Angeles Classification | None | 6 (66.7%) |
|  | B | 3 (33.3%) |
| Postoperative PPI use at 1 year |  | 4 (44.4%) |

**Suppl. Table 1:** GERD Assessment using pH-impendance and HR-manometry.

Values are medians (interquartile ranges (IQR)) or number (percentages), respectively.

Abbreviations:EGD, esophago-gastro-duodenoscopy; GERD, gastroesophageal reflux disease; PPI, proton pump inhibitor;

| Pat. | Pr. BMI | Po. BMI | %EWL | Pr. EAT | Pr. RefEp | Po. EAT | Po. RefEp |
| --- | --- | --- | --- | --- | --- | --- | --- |
| 1 | 50.10 | 36.36 | 68.34 | 89.4 | 66 |  |  |
| 2 | 42.61 | 32.31 | 81.65 | 3.1 | 19 | 86.8 | 36 |
| 3 | 39.67 | 29.75 | 102.56 | 10.5 | 32 | 3.1 | 44 |
| 4 | 60.48 | 47.23 | 43.46 | 6.9 | 45 | 1.1 | 99 |
| 5 | 38.82 | 29.41 | 106.67 |  |  | 1.2 | 34 |
| 6 | 44.06 | 24.84 | 136.72 | 8.5 | 44 | 13.8 | 73 |
| 7 | 35.47 | 23.24 | 223.68 | 7.3 | 37 | 1.9 | 19 |
| 8 | 45.17 | 31.99 | 86.86 | 4.8 | 58 | 14.7 | 120 |
| 9 | 49.95 | 36.00 | 69.90 | 2.9 | 59 | 0.4 | 132 |

**Suppl. Table 2a:** Patient individual GERD characteristics of pH impendance manimetry results, in accordance with Lyon Consensus 2018 Guidelines.

| Pat. | Pr. GEJ DI | Intra. GEJ DI | Po. GEJ DI | Pr. Pylorus DI | Intra. Pylorus DI | Po. Pylorus DI |
| --- | --- | --- | --- | --- | --- | --- |
| 1 | 4.0 | 12.2 | 5.3 | 5.6 | 10.3 | 21.4 |
| 2 | 4.3 | 2.9 | 6.0 | 6.3 | 3.8 | 12.2 |
| 3 | 1.1 | 4.7 | 1.7 | 12.5 | 9.9 | 14.0 |
| 4 | 2.6 | 8.9 | 4.7 | 9 | 2.3 | 17.0 |
| 5 | 0.5 | 1.4 | 2.9 | 3.8 | 1.4 | 5.3 |
| 6 | 1.2 |  | 1.0 | 12.4 |  | 9.2 |
| 7 | 0.5 | 1.9 | 2.8 |  | 4.3 | 22.3 |
| 8 | 2.6 |  | 5.6 | 4 |  | 6.0 |
| 9 | 1.4 |  | 0.9 | 4.1 |  |  |

**Suppl. Table 2b:** Patient individual pre, intra and postoperative assessment of gastroesophageal junction and pylorus characteristics at 40mL filling volume using EndoFlip^TM^.

Abbreviations: %EWL, percentage of excess weight loss; BMI, body mass index; DI, distensibility index [mm^2^/mmHg]; EAT, distal esophageal acid (ph<4) exposure time [%]; GEJ, gastroesophageal junction; intra., intraoperative; Pat., patient; Po., postoperative; Pr., preoperative; RefEp, total number of reflux episodes (acid and non-acid)
